# Vaccination with Recombinant *Mycobacterium tuberculosis* PknD Attenuates Bacterial Dissemination to the Brain in Guinea Pigs

**DOI:** 10.1371/journal.pone.0066310

**Published:** 2013-06-11

**Authors:** Ciaran Skerry, Supriya Pokkali, Michael Pinn, Nicholas A. Be, Jamie Harper, Petros C. Karakousis, Sanjay K. Jain

**Affiliations:** 1 Center for Tuberculosis Research, Johns Hopkins University School of Medicine, Baltimore, Maryland, United States of America; 2 Department of Pediatrics, Johns Hopkins University School of Medicine, Baltimore, Maryland, United States of America; 3 Department of Medicine, Johns Hopkins University School of Medicine, Baltimore, Maryland, United States of America; 4 Physical and Life Sciences Directorate, Lawrence Livermore National Laboratory, Livermore, California, United States of America; 5 Center for Infection and Inflammation Imaging Research, Johns Hopkins University School of Medicine, Baltimore, Maryland, United States of America; Institut de Pharmacologie et de Biologie Structurale, France

## Abstract

**Background:**

We have previously identified *Mycobacterium tuberculosis* PknD to be an important virulence factor required for the pathogenesis of central nervous system (CNS) tuberculosis (TB). Specifically, PknD mediates bacillary invasion of the blood-brain barrier, which can be neutralized by specific antisera, suggesting its potential role as a therapeutic target against TB meningitis.

**Methodology/Principal Findings:**

We utilized an aerosol challenge guinea pig model of CNS TB and compared the protective efficacy of recombinant *M. tuberculosis* PknD subunit protein with that of *M. bovis* BCG against bacillary dissemination to the brain. BCG vaccination limited the pulmonary bacillary burden after aerosol challenge with virulent *M. tuberculosis* in guinea pigs and also reduced bacillary dissemination to the brain (P = 0.01). PknD vaccination also offered significant protection against bacterial dissemination to the brain, which was no different from BCG (P>0.24), even though PknD vaccinated animals had almost 100-fold higher pulmonary bacterial burdens. Higher levels of PknD-specific IgG were noted in animals immunized with PknD, but not in BCG-vaccinated or control animals. Furthermore, pre-incubation of *M. tuberculosis* with sera from PknD-vaccinated animals, but not with sera from BCG-vaccinated or control animals, significantly reduced bacterial invasion in a human blood-brain barrier model (P<0.01).

**Conclusion:**

Current recommendations for administering BCG at birth are based on protection gained against severe disease, such as TB meningitis, during infancy. We demonstrate that vaccination with recombinant *M. tuberculosis* PknD subunit offers a novel strategy to protect against TB meningitis, which is equivalent to BCG in a guinea pig model. Moreover, since BCG lacks the PknD sensor, BCG could also be boosted to develop a more effective vaccine against TB meningitis, a devastating disease that disproportionately affects young children.

## Introduction

Central nervous system (CNS) tuberculosis (TB) is a serious, often fatal disease that predominantly affects young children [1], [2], [3]. Two major forms of CNS TB include meningitis which accounts for 0.5–1% of all TB disease, and intra-cranial tuberculomas, which on a global level account for up to 40% of ‘brain tumors’ [2], [4]. Co-infection with HIV not only increases the risk of development of CNS TB [5], [6], but also leads to a much higher case-fatality rate [7], [8]. Non-specific clinical presentation, poor diagnostics, and delays in instituting appropriate TB treatment (drug susceptibility tests take up to 10 weeks), complicate CNS TB leading to severe, irreversible neurological damage and high mortality. Treatment of CNS TB becomes even more challenging in the age of multi-drug (MDR), extensively-drug (XDR) and totally-drug resistant (TDR) strains of *Mycobacterium tuberculosis* [9]. Moreover, several second line TB drugs have limited CNS penetration. Since clinical outcomes (even with appropriate treatment) after the onset of TB meningitis are extremely poor [10], [11], [12], developing preventive strategies against CNS TB is a high priority.

We have previously characterized *M. tuberculosis* PknD, a transmembrane protein with an extracellular surface exposed (sensor) domain [13], to be an important virulence factor required for CNS TB in animal models [14], [15]. We have also demonstrated that the *M. tuberculosis* PknD sensor domain is sufficient to trigger invasion of human brain microvascular endothelia, which are the primary components of the blood-brain barrier (BBB) protecting the brain. Moreover, invasion of human brain microvascular endothelia could be neutralized by pre-incubation of bacteria with PknD (sensor)-specific antisera [14]. Since CNS TB and TB meningitis develop due to extrapulmonary hematogenous dissemination of bacilli, these data suggest that the extracellular (sensor) surface exposed domain of *M. tuberculosis* PknD could be targeted to protect against TB meningitis. In this study, we utilized a guinea pig model with reliable bacillary dissemination to the brain following aerosol challenge with *M. tuberculosis* [16]. This model was used to compare the protective efficacy of recombinant *M. tuberculosis* PknD sensor protein with that of BCG *against M.* tuberculosis dissemination to the brain.

## Materials and Methods

### Ethics Statement

All animal procedures have been approved by the ethics committee of Johns Hopkins University.

### Vaccination

Recombinant *M. tuberculosis* PknD sensor protein was expressed as described previously [14]. Briefly, the coding sequence for PknD amino acid residues 403–664 was cloned into pDEST17 (6x N-terminal his-tag) using the Gateway cloning system (Invitrogen, Grand Island, NY). Expression of PknD protein was induced using 0.1% L-arabinose at 37°C in BL21-AI *E. coli*. PknD protein was purified by SDS-PAGE. Vaccinations were performed subcutaneously at 10-, 7-, and 4-weeks prior to aerosol challenge. Animals were divided into four groups: negative controls, injected with equal volumes of PBS; positive controls, injected with a single dose of *M. bovis* BCG (Danish strain 1331) [5x10^4^ colony-forming units (CFU) in a total volume of 200 µl] 10 weeks prior to aerosol challenge; adjuvant group injected with 20 µg of dimethyldioctadecylammonium bromide [DDA (Sigma-Aldrich, St. Louis, MO)]; and PknD group injected with 20 µg of recombinant *M. tuberculosis* PknD sensor protein in combination with 20 µg of DDA. After vaccination, but prior to infection, blood was also harvested to obtain sera.

### Animal infection

Hartley guinea pigs (200–250g) (Charles River, Wilmington, MA) were aerosol-infected with frozen titrated bacterial stocks of *M. tuberculosis* CDC1551 (grown to OD_600_ of 1.0), using the Madison chamber (University of Wisconsin, Madison, WI) [17]. Three animals were sacrificed one day after infection to determine implantation. Animals were sacrificed at 4- and 6-weeks post-infection to determine bacillary burden. Lungs, and brains were removed aseptically, homogenized and plated on Middlebrook 7H11 agar plates to determine CFU [16]. The entire brains were homogenized in 10 ml of PBS. One ml was diluted into 9 ml (10 fold dilution) and plated separately. The entirety of the remaining 9 ml was plated and used to determine CFU. 2-Thiophenecarboxylic acid hydrazide (TCH) agar (Sigma-Aldrich) selectively allows growth of *M. tuberculosis*, but inhibits BCG. Therefore, TCH plates were used to determine *M. tuberculosis* CFU for tissues obtained from BCG vaccinated animals. Four guinea pigs per group were sacrificed at each time-point, except for the DDA group where only three animals were sacrificed at the 6-weeks time-point. By 6-weeks post infection, animals displayed greater morbidity than anticipated, and therefore the decision was made to terminate the study at the 6-weeks post-infection time-point. For mortality data, each death represents an animal found dead in the cage, or deemed to be too sick to remain in the study (based on akinesia, labored breathing and malaise) by the veterinary care staff. Organs from animals found dead in the cages were not harvested for CFU.

### Cell proliferation and IFN-γ assays

Guinea pig splenocytes were isolated by disaggregation of spleens, viability assayed using trypan blue (Sigma-Aldrich, USA) and cultured in triplicate with either 10^6^ CFU of heat-inactivated *M. tuberculosis* CDC1551, 10 µg of recombinant PknD sensor protein, or culture media alone (control), at a concentration of 1×10^6^ cells/ml in a total volume of 200 µl. After 72 h of incubation, culture supernatants were sampled for determination of IFN-γ concentrations using an anti-guinea pig IFN- using an immunosorbent kit (Uscn Life Science Inc., Wuhan, China) as described previously [18]. Cell proliferation was measured using the Cell Proliferation Reagent WST-1 (Roche, Indianapolis, IN) per manufacturer's instructions. Briefly, cells grown in a 96 well plate were incubated for 1 hour at 37°C, 5% CO_2_ with 20 µl of the WST-1 reagent, before absorbance was measured at 450 nm. Data were normalized to the culture media alone (control). Data are presented as stimulation index, defined as the proliferation observed in test samples divided by that seen in control samples (culture media alone).

### Determination of IgG antibodies

At each time-point, 10 ml of blood was obtained via cardiac puncture. Blood was allowed to clot overnight at 4°C and serum isolated by centrifugation (4000 RPM, 20 minutes, 4°C). Sera were processed in a 96-well ELISA plates (Thermo Scientific). Plates were coated overnight with recombinant *M. tuberculosis* PknD (1 µg) in carbonate coating buffer (3.03g Na_2_CO_3_, 6g NaHCO_3_ in 1L dH_2_0, pH 9.6) sensor protein or 10 µl of heat-inactivated *M. tuberculosis* CDC1551 (10^7^ CFU/ml), blocked with 1% bovine serum albumin BSA (weight/volume) (Sigma-Aldrich) and washed with 0.1% Tween 20 (Sigma-Aldrich) and incubated with 1000-fold diluted guinea pig sera for 1 hour at 37°C. Following washing, horseradish peroxidase (HRP) conjugated IgG detection antibody (BD, Franklin Lakes, NJ) was added and incubated for 1 hour at 37°C, washed again with subsequent addition of the HRP substrate 3, 3′, 5, 5′-tetramethylbenzidine (TMB) (Sigma-Aldrich). The reaction was stopped after 20 minutes using 1 M H_2_SO_4_ and absorbance measured at 450 nm. Sera from three animals from each group were tested. Each assay was performed in triplicate.

### *In vitro* neutralization assays

*M. tuberculosis* CDC1551 were pre-incubated with guinea pig sera (1:1250 dilution) for 60 minutes. Bacteria were subsequently washed in PBS and used for invasion assays with primary human brain microvascular endothelial cells (HBMEC) as described previously [14]. Sera from three animals from each group were tested. Each assay was performed in triplicate.

### Statistical analysis

Comparison between groups were performed using a one tail distribution, two sample unequal variance Student's t test in Excel 2007 (Microsoft) except for CFU data where a one tail Mann-Whitney U test (http://elegans.som.vcu.edu/~leon/stats/utest.html) and stimulation indices where a One-way ANOVA with Bonferroni's post-hoc test (Prism 5 version 5.01, GraphPad software, San Diego, CA) were utilized. Data are presented on a linear scale as mean ± standard deviation (SD) except for CFU, where a logarithmic scale (mean±SD) has been used.

## Results

### PknD vaccination protects against *M. tuberculosis* dissemination to the brain

**Figure 1:**
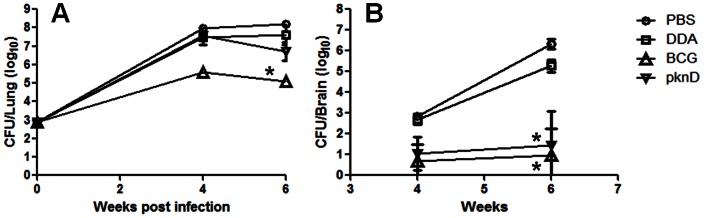
PknD vaccination protects against *M.* *tuberculosis* dissemination to the brain. Colony-forming units (CFU) in the lungs (panel A) and brains (panel B) of guinea pigs vaccinated with BCG (triangle), recombinant PknD (inverted triangle), DDA (adjuvant alone) (square) or PBS (unvaccinated controls) (circle), 4- and 6-weeks after an aerosol challenge with *M. tuberculosis* are shown. BCG vaccination limited the pulmonary bacillary load (P = 0.01) and also significantly reduced the bacillary burden in the brains after aerosol challenge with virulent *M. tuberculosis* (P = 0.01). While PknD vaccination did not limit bacillary growth in the lungs, it offered significant protection against bacillary dissemination to the brain, which was no different from BCG (P>0.24), even though the PknD vaccinated animals had almost 100-fold higher bacterial burdens in the lungs. Four guinea pigs per group were sacrificed at each time-point, except for the DDA group, for which only three animals were sacrificed 6-weeks after aerosol challenge. Data are presented on a logarithmic scale as mean±standard deviation.

Groups of PknD, BCG vaccinated, unvaccinated (PBS) and animals administered adjuvant only (DDA) were challenged via aerosol with virulent *M. tuberculosis* and the bacillary burden in the lungs and brains was assessed 4- and 6-weeks after infection. Pulmonary implantation one day after aerosol infection was 2.90±0.06 log_10_ CFU. Compared with unvaccinated animals (PBS), BCG vaccination significantly limited the pulmonary CFU burdens at both 4-weeks (5.61±0.12 versus 7.99±0.12 log_10_ CFU; P = 0.01) and 6-weeks (5.09±0.16 versus 8.20±0.17 log_10_ CFU; P = 0.01) after the aerosol challenge. However, the pulmonary bacillary burdens in PknD- and DDA-vaccinated groups were similar to the unvaccinated (PBS) group (Figure 1A).

Whole brains were also harvested from each animal to measure *M. tuberculosis* dissemination to the CNS. Compared with unvaccinated animals (PBS), BCG vaccination significantly reduced the bacterial burdens in the brain at both 4-weeks (0.70±0.40 versus 2.84±0.06 log_10_ CFU; P = 0.01) and 6-weeks (0.97±1.27 versus 6.32±0.25 log_10_ CFU; P = 0.01) after infection. PknD vaccination also significantly reduced the bacterial burdens in the brain at both 4-weeks (1.05±0.40 versus 2.84±0.06 log_10_ CFU; P = 0.01) and 6-weeks (1.44±1.66 versus 6.32±0.25 log_10_ CFU; P = 0.01) after infection (Figure 1B) and this protection was no different from that offered by BCG (P>0.24), even though the PknD vaccinated animals had almost 100-fold higher bacterial burdens in the lungs. These data suggest that vaccination with *M. tuberculosis* PknD sensor protects against bacterial dissemination to the brain. Interestingly, a trend for lower mortality was noted in both the BCG (25%; 1 of 4) or PknD (20%; 1 of 5) vaccinated groups compared with the control groups [PBS (71%; 5 of 7) or DDA (100%; 4 of 4), where a much higher mortality was noted.

### PknD vaccination induces specific IFN-γ and IgG responses

**Figure 2:**
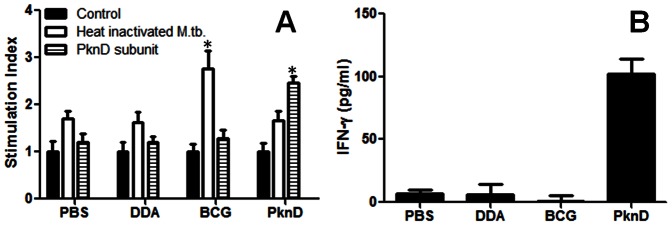
PknD vaccination induces specific IFN-γ and IgG responses. Splenic recall assays were performed on splenocytes from guinea pigs vaccinated with BCG, recombinant PknD, DDA (adjuvant alone) or PBS (unvaccinated controls). The WST-1 system was then used to measure proliferation after stimulation heat-inactivated *M. tuberculosis* (white bars), recombinant PknD subunit (striped bars) or control with culture media alone (black bars) (panel A). Significant cellular proliferation in response to PknD subunit was only observed in splenocytes from PknD vaccinated animals (P = 0.002). Similarly, significant proliferation in response to heat-inactivated *M. tuberculosis* was only observed in splenocytes from BCG vaccinated animals (P = 0.01). Supernatants from these assays were also used to measure the IFN-γ levels (panel B). Consistent with proliferation data, only animals vaccinated with PknD (P<0.01) generated appreciable levels of IFN-γ in response to the PknD subunit protein (Figure 2B). Splenocytes from three animals from each group were tested. Each assay was performed in triplicate. Data are presented on a linear scale as mean±standard deviation.

To determine the ability of PknD to induce splenocyte proliferation, cells were isolated from the spleens of animals from each group. Cell preparations were then exposed to culture media (control), heat-inactivated *M. tuberculosis* or recombinant PknD subunit in the WST-1 assay, which measures formazan formation by the mitochondrial dehydrogenase of viable cells and provides a sensitive and accurate nonradioactive method to measure cell proliferation [19], [20], [21] (Figure 2A). Significant cellular proliferation in response to PknD subunit was only observed in splenocytes from PknD vaccinated animals (P = 0.002). Similarly, significant proliferation in response to heat-inactivated *M. tuberculosis* was only observed in splenocytes from BCG vaccinated animals (P = 0.01). Supernatants from these stimulation assays were also harvested to determine IFN-γ levels. Consistent with proliferation data, only animals vaccinated with PknD (P<0.01) generated appreciable levels of IFN-γ in response to the PknD subunit protein (Figure 2B).

### PknD vaccinated animals have high levels of PknD-specific IgG antibodies

**Figure 3:**
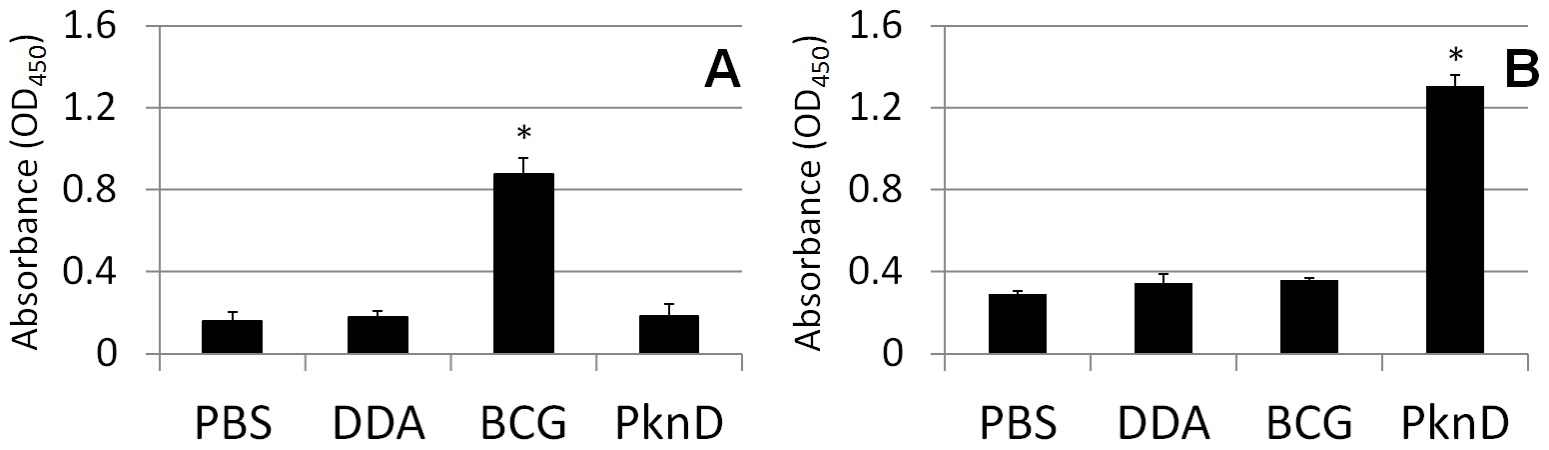
*M. tuberculosis* PknD-specific IgG in vaccinated guinea pigs. *M. tuberculosis*, blood was harvested from each animal to obtain sera. The levels of IgG antibodies reactive to heat-inactivated *M. tuberculosis* or to recombinant PknD sensor were determined for each group. While sera from BCG-vaccinated animals demonstrated high levels of IgG antibodies reactive to heat-inactivated *M. tuberculosis* (P<0.01) (panel A), only sera from PknD-vaccinated animals showed high levels of IgG antibodies reactive to PknD (P<0.01) (panel B). Sera from three animals from each group were tested. Each assay was performed in triplicate. Data are presented on a linear scale as mean±standard deviation.

After completion of vaccination, but just prior to aerosol challenge with *M. tuberculosis*, blood was harvested from each animal to obtain sera. The levels of IgG antibodies reactive to heat-inactivated *M. tuberculosis* or to recombinant PknD sensor were determined for each group. As expected, only sera from BCG-vaccinated animals had high levels of IgG antibodies reactive to heat-inactivated *M. tuberculosis* (P<0.01) (Figure 3A). In contrast, only sera from PknD-vaccinated animals had high levels of IgG antibodies reactive to PknD (P<0.01) (Figure 3B).

### Pre-incubation of *M. tuberculosis* with sera from PknD-vaccinated guinea pigs reduces invasion of brain microvascular endothelia

**Figure 4:**
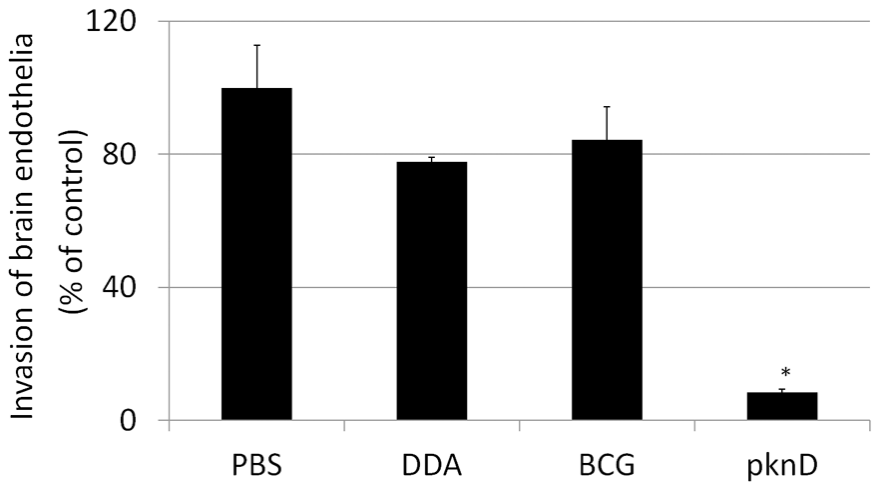
Pre-incubation of *M.* *tuberculosis* with sera from PknD-vaccinated guinea pigs reduces invasion of brain microvascular endothelia: Human brain microvascular endothelia are the primary components of the blood-brain barrier and protect the CNS from the systemic circulation. Brain microvascular endothelial invasion of *M. tuberculosis* pre-incubated with sera from each vaccinated group normalized to the unvaccinated animals (PBS) is shown. While sera from the BCG vaccinated animals was not protective, pre-incubation of *M. tuberculosis* with sera from PknD-vaccinated guinea pigs significantly reduced (>10 fold) invasion of brain microvascular endothelia (P<0.01). Sera from three animals from each group were tested. Each assay was performed in triplicate. Data are presented on a linear scale as mean±standard deviation.

Human brain microvascular endothelia are the primary components of the blood-brain barrier and protect the central nervous system [22]. We have previously demonstrated that invasion of human brain microvascular endothelia by *M. tuberculosis* can be neutralized by PknD (sensor)-specific antiserum [14]. To further assess whether sera from PknD-vaccinated animals were indeed protective, we tested whether pre-incubation of *M. tuberculosis* with guinea pig sera reduced invasion of the brain microvascular endothelia. Figure 4 demonstrates the invasion of *M. tuberculosis* pre-incubated with sera from each vaccinated group, normalized to the unvaccinated animals (PBS). While sera from the BCG-vaccinated animals was not protective, pre-incubation of *M. tuberculosis* with sera from PknD-vaccinated guinea pigs significantly reduced (>10 fold) invasion of brain microvascular endothelia (P<0.01). Collectively, these data suggest that PknD vaccinated animals produced a robust PknD-specific IgG response that also prevents invasion of brain microvascular endothelia.

## Discussion

BCG is the only licensed vaccine against TB and is recommended for administration to all newborns (at birth) in countries with a high TB burden by the World Health Organization (WHO) [23]. However, the protection offered by BCG for adult pulmonary TB is highly variable (0–80%) [24], [25], and the current WHO recommendation is based on the ability of BCG to protect against severe forms of TB, such as TB meningitis, during infancy [23]. BCG works by the induction of T-cell immune responses that limit *M. tuberculosis* burden at the site of primary infection (mostly lungs). Since the magnitude of the pulmonary bacterial burden determines extrapulmonary dissemination, the reduction in the pulmonary burden due to BCG, decreases extrapulmonary hematogenous dissemination of bacteria and subsequent risk of developing CNS TB and meningitis [23]. Unfortunately, protection offered by BCG against TB meningitis is also quite variable (52–100%) [26], [27], [28], [29]. BCG is not well-defined antigenically, and the several different BCG strains in clinical use, offer variable levels of protection [30]. Moreover, BCG is a live vaccine, and therefore may be unsuitable for immunosuppressed infants especially in the setting of HIV [31]. An additional limitation is that BCG confounds the interpretation of the tuberculin skin test, which would not apply to subunit vaccines. For these reasons, a new, preferably acellular, alternative to BCG vaccination would be ideal. To this end, several acellular candidates exist [32], [33], [34], though none have explored the ability to prevent CNS TB.

We have previously demonstrated that mutants deficient in *M. tuberculosis pknD* exhibit CNS attenuation, with no corresponding defect in lung tissues in two different animal models of TB (mice, guinea pigs) [14], [15]. We have also demonstrated that *M. tuberculosis pknD* is required for invasion of brain microvascular endothelia (primary components of the BBB) and that invasion by wild-type *M. tuberculosis* can be neutralized by pre-incubation with PknD (sensor)-specific antisera [14]. Interestingly, based on gene sequence analyses, pathogenic strains of *M. tuberculosis* produce the full PknD. However, a frame shift mutation in the *pknD* homolog in BCG, results in a predicted truncated protein without the C-terminal sensor domain [35]. We therefore hypothesized that vaccination strategies targeting *M. tuberculosis* PknD (sensor) can serve as novel methods to protect against CNS TB and TB meningitis. To test this hypothesis, we utilized an aerosol challenge guinea pig model, with hematogenous bacterial dissemination to the brain [16]. Following vaccination, all animal groups were challenged, via aerosol, with virulent *M. tuberculosis* and the bacterial burden assessed 4- and 6-weeks after infection. Consistent with prior data [23], [36], BCG vaccination significantly limited the pulmonary bacterial burden after the aerosol challenge, and also significantly reduced bacterial dissemination to the brain. However, while vaccination with PknD did not affect the bacterial burden in the lungs at 4-weeks and only modestly decreased bacterial burden in the lungs at 6-weeks, it nonetheless significantly reduced bacterial dissemination to the brain at both time-points measured. While the modest decrease in pulmonary bacterial burden in the PknD vaccinated group at 6-weeks could have partially contributed to the decreased bacterial dissemination to the CNS, highly significant differences were noted in bacterial dissemination to the CNS even at 4-weeks, when the pulmonary bacterial burden in the PknD vaccinated groups were no different than the controls. In fact, even though the PknD vaccinated animals had an almost 100-fold higher bacterial burden in the lungs, PknD offered protection similar to BCG at either time-point evaluated in this study (P>0.24). Though these data are consistent with our prior observation of the role of *M. tuberculosis* PknD in the pathogenesis of CNS disease (with no corresponding role in the lung tissues), it should be noted that other extrapulmonary sites such as spleen were not evaluated in the current study. Therefore, the possibility of a more generalized extrapulmonary protection offered by PknD vaccination cannot be ruled out. Interestingly, a trend for lower mortality was noted in both the BCG or PknD vaccinated groups. However, these data were not statistically significant, due to the low number of animals in this study since determination of mortality rates amongst the different vaccination groups was not one of the end-points.

While previous efforts at developing TB vaccines have focused on the generation of cellular responses; several studies have demonstrated the role of antibodies in protection against pulmonary TB [37], [38], [39], although none have explored the ability to prevent CNS TB. Since TB meningitis develops subsequent to hematogenous dissemination of bacteria [40], and the surface exposed PknD sensor is required for invading the BBB that protects the CNS from the systemic circulation [13], [14], [41], [42], we hypothesized that antibody-mediated humoral immunity against PknD could be protecting against brain dissemination in the PknD vaccinated animals. Our data indicate that a robust PknD-specific IFN-γ response and higher levels of PknD-specific IgG were noted in animals vaccinated with PknD, but not in those vaccinated with BCG or control animals. Furthermore, we were also able to demonstrate that pre-incubation of *M. tuberculosis* with sera from PknD- vaccinated, but not sera from BCG-vaccinated or control animals could neutralize bacterial invasion of brain microvascular endothelia. Collectively, these data indicate that components of sera, likely PknD-specific IgG, protect against bacillary dissemination to the brain in PknD-vaccinated animals. One of the limitations of the current study is that we utilized unpurified sera, as opposed to purified PknD-specific antibodies for the inhibition assays. However, high dilutions of sera were utilized to minimize any non-specific interactions. In addition, at this time, we do not know the antibody subclass that is potentially involved in neutralizing bacterial invasion of brain microvascular endothelia. Finally, guinea-pigs are outbred, which precluded the possibility of performing passive transfer experiments, which may have further strengthened our data. This limitation may be overcome by utilizing inbred species such as mice, and may be pursued in future experiments.

Since the major reason for BCG vaccination at birth is for its protective effect against TB meningitis during infancy [23], vaccination with the PknD subunit could offer similar, but more consistent protection against CNS TB, especially for immunosuppressed infants in the setting of HIV. Moreover, since BCG does not express the sensor domain of PknD [35], current BCG vaccines could also be boosted by either developing a recombinant strain that expresses PknD, or by concomitant vaccination with PknD subunit in combination with BCG.

In summary, we have demonstrated that vaccination with recombinant PknD sensor protein protects against bacterial dissemination to the brain in a guinea pig model. Moreover, this protection is likely mediated by humoral IgG responses against PknD (sensor) that prevent the invasion of brain microvascular endothelia. These data are expected to help in developing better vaccines against TB meningitis, a devastating disease that disproportionately affects young children.
